# Supplementary material for: Coelastrella terrestris for Adonixanthin Production: Physiological Characterization and Evaluation of Secondary Carotenoid Productivity
Source: Mar Drugs. 2022 Feb 26;20(3):175. doi: 10.3390/md20030175 (PMC8954916; doi:10.3390/md20030175)
Supplement: Supplementary file 1 [file marinedrugs-20-00175-s001.zip › marinedrugs-1574177-Supplementary.pdf]

Supplementary Material

Coelastrella terrestris for Adonixanthin Production: Physiological Characterization and Evaluation of Secondary Carotenoid Productivity

Philipp Doppler, Ricarda Kriechbaum, Julian Kopp, Maria Käfer, Daniel Remias and Oliver Spadiut

Table S1. Biomass concentration and volumetric and specific pigment and secondary carotenoid concentrations of 1.25 L lab-scale photobioreactor cultivations of *C. terrestris*.

	BBM			2N-BBM		
	**Glucose**	**Acetate**	**Control**	**Glucose**	**Acetate**	**Control**
**DCW (g·L^{-1})**	2.87	2.40	3.78	3.82	3.49	4.00
**SC (mg·L^{-1})**	5.53	1.99	2.80	4.88	6.39	6.14
**SC (mg·g^{-1})**	1.93	0.83	0.75	1.28	1.83	1.54
**PC (mg·L^{-1})**	2.23	1.02	1.79	4.19	2.90	4.65
**PC (mg·g^{-1})**	0.78	0.42	0.47	1.10	0.83	1.16
**Chl (mg·L^{-1})**	9.39	5.41	9.22	12.18	12.29	20.55
**Chl (mg·g^{-1})**	3.28	2.26	2.44	3.19	3.52	5.14
**Astaxanthin (mg·L^{-1})**	2.25	1.00	1.51	2.44	2.65	2.66
**Astaxanthin (mg·g^{-1})**	0.78	0.42	0.40	0.64	0.76	0.67
**Adonixanthin (mg·L^{-1})**	1.46	0.57	0.73	1.54	1.96	1.64
**Adonixanthin (mg·g^{-1})**	0.51	0.24	0.19	0.40	0.56	0.41
**Canthaxanthin (mg·L^{-1})**	1.58	0.41	0.60	0.77	1.58	1.68
**Canthaxanthin (mg·g^{-1})**	0.55	0.17	0.16	0.20	0.45	0.42
**Echinenone (mg·L^{-1})**	0.24	0.02	0.00	0.14	0.20	0.16
**Echinenone (mg·g^{-1})**	0.08	0.01	0.00	0.04	0.06	0.04

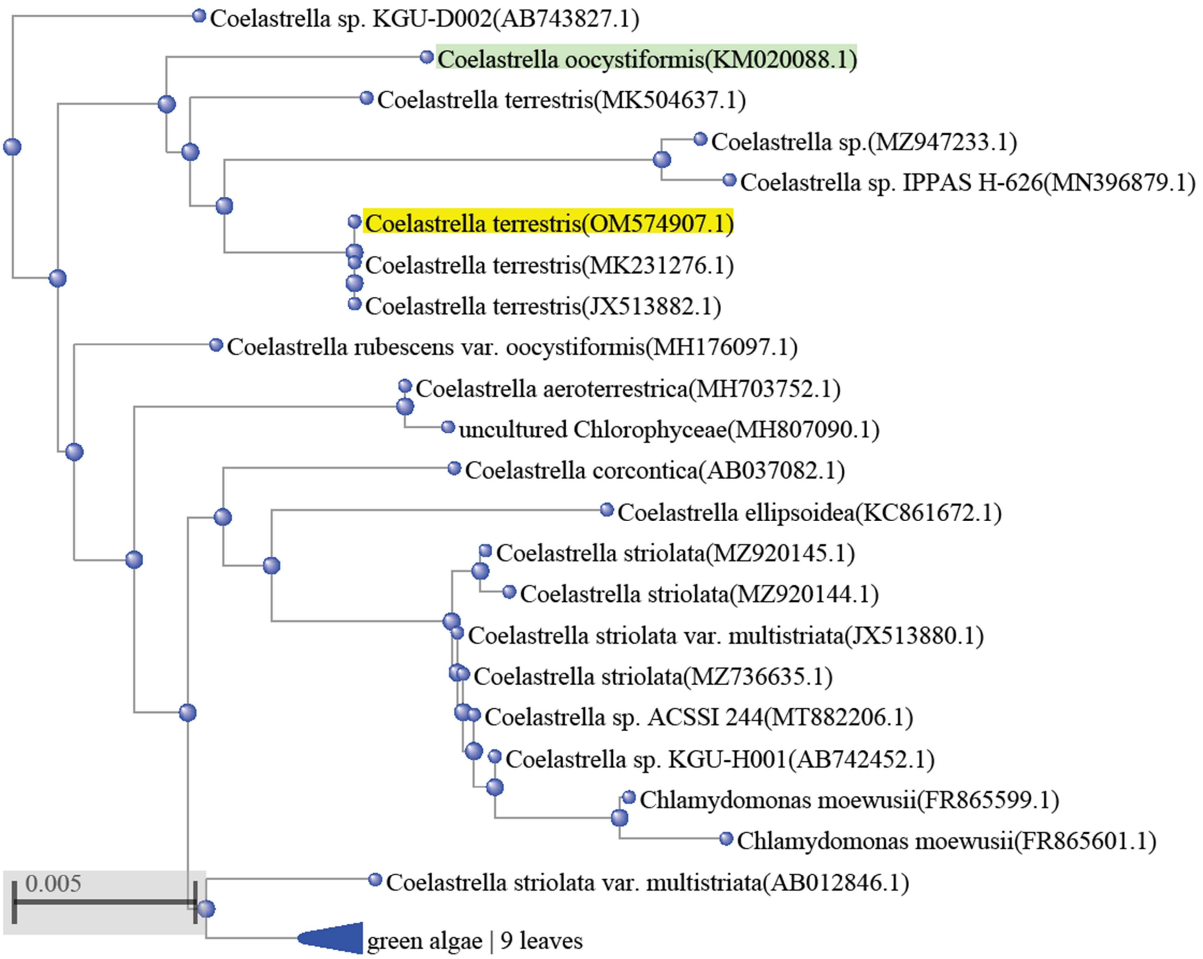

Figure S1. Phylogenetic tree of the strain *Coelastrella terrestris* WP154.2 (highlighted in yellow, accession number OM574907.1). The analysis based on the 1067 nucleotides of the nuclear 18S rDNA fragment. It was drawn by the NCBI tool for showing distances to related species by the neighbor joining tree method. Type strains are highlighted in green. Closest relatives of WP154.2 were *Coelastrella terrestris* strain CCALA 476 (accession JX513882.1) and *Coelastrella terrestris* strain KZ-5-4-9 (accession number MK231276.1).

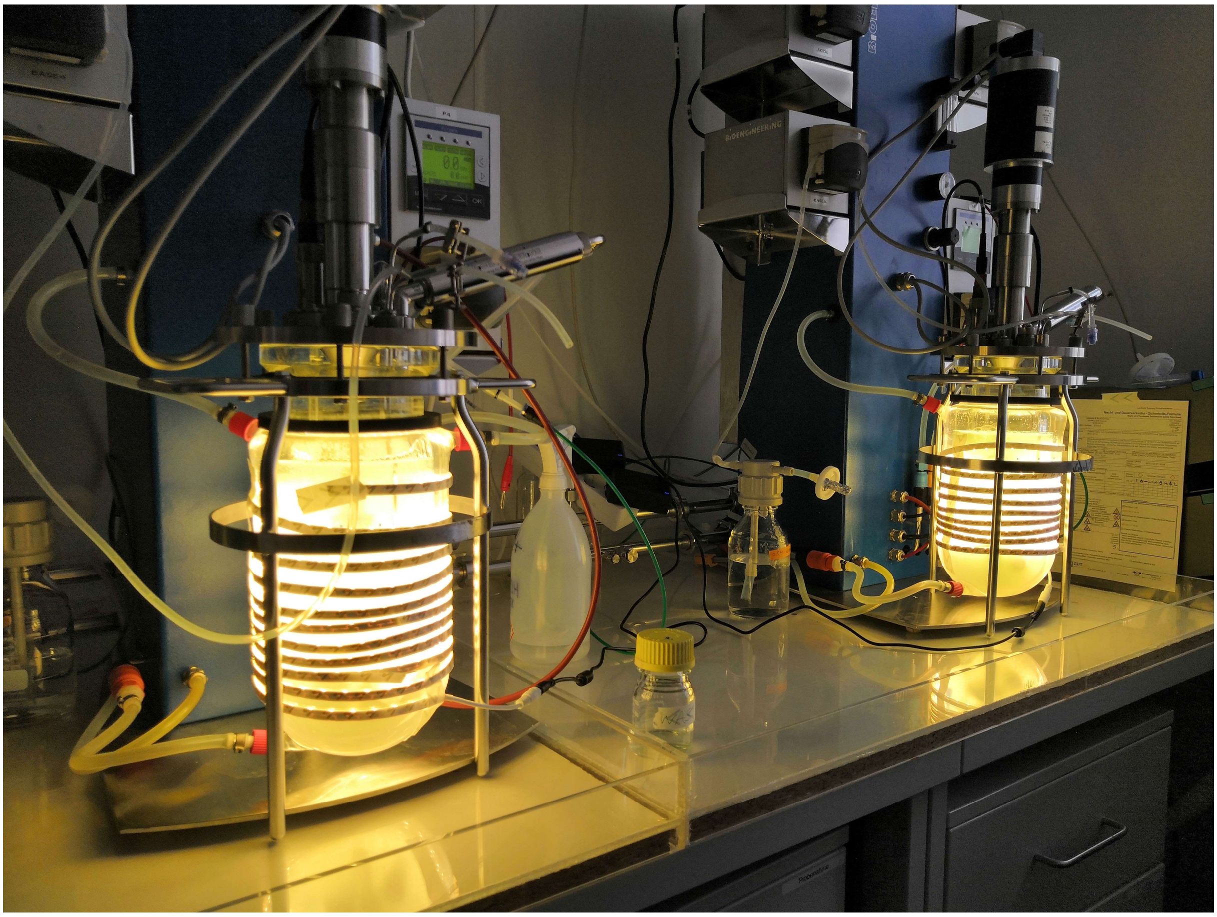

Figure S2. The externally illuminated stirred tank photobioreactor (PBR) system used for 1.25 L lab-scale cultivation of *C. terrestris* WP154.2.
